# Telemedicine-enabled Accelerated Discharge of Patients Hospitalized with COVID-19 to Isolation in Repurposed Hotel Rooms

**DOI:** 10.1164/rccm.202004-1238OE

**Published:** 2020-08-15

**Authors:** Teresa Bruni, Ajit Lalvani, Luca Richeldi

**Affiliations:** ^1^ Unità Operativa Complessa di Pneumologia, Fondazione Policlinico Universitario A. Gemelli–Istituto di Ricovero e Cura a Carattere Scientifico, Università Cattolica del Sacro Cuore, Roma, Italy; and; ^2^ National Institute for Health Research Health Protection Research Unit in Respiratory Infections, National Heart and Lung Institute, Imperial College London, London, United Kingdom

## The Need to Optimize Efficient Use of Hospital Beds

Healthcare facilities, especially hospitals, are facing unprecedented degrees of demand on account of large and growing numbers of patients with coronavirus disease (COVID-19) requiring hospital admission. As recently witnessed in northern Italy (1), when patient inflows exceed outflows, hospitals will be overwhelmed; the use of resources must be optimized to avoid this tipping point. Our viewpoint is that expansion of high-dependency unit and ICU capacity is best achieved by upgrading existing lower-dependency beds rather than by building *de novo* field hospitals from scratch without the integrated complex infrastructure required for hospital-based care, including oxygen. This approach identifies a sort of “reverse triage,” which has already been used in other types of catastrophic events to increase the capacity of ICUs and emergency departments (2), and could solve several problems, including diversion of mechanical ventilators and highly trained staff away from existing hospitals that are trying to expand their capacities to meet the surge in demand.

This proposed model requires accelerating discharge of low-dependency patients into isolation. Patients who can be discharged faster to release low-dependency hospital beds are primarily recovering from COVID-19 and awaiting a PCR-negative swab result before leaving isolation rooms. These patients often cannot be discharged to their homes for keeping self-isolation, for at least one of two reasons. First, because of the risk of infecting their cohabitants, achieving proper isolation in homes with more than one resident is often logistically extremely difficult, and cohabitants often include highly susceptible, older people or those with underlying conditions, especially if the patient resides in a care home. Even patients who are homeless cannot self-isolate. Second, patients may require ongoing monitoring of vital signs before being fit for discharge. Telemonitoring guaranteed during the period of infection makes the hospital physician more comfortable to quickly discharge a patient who recently had pneumonia and low Sa_O_2__. Our model enables these patients to continue isolation safely in a medically monitored environment, avoiding the adverse clinical consequences of discharging still-infectious individuals to their homes and care homes.

## Repurposing Empty Hotel Rooms to Enable Isolation in a Monitored Environment

**Figure 1.**
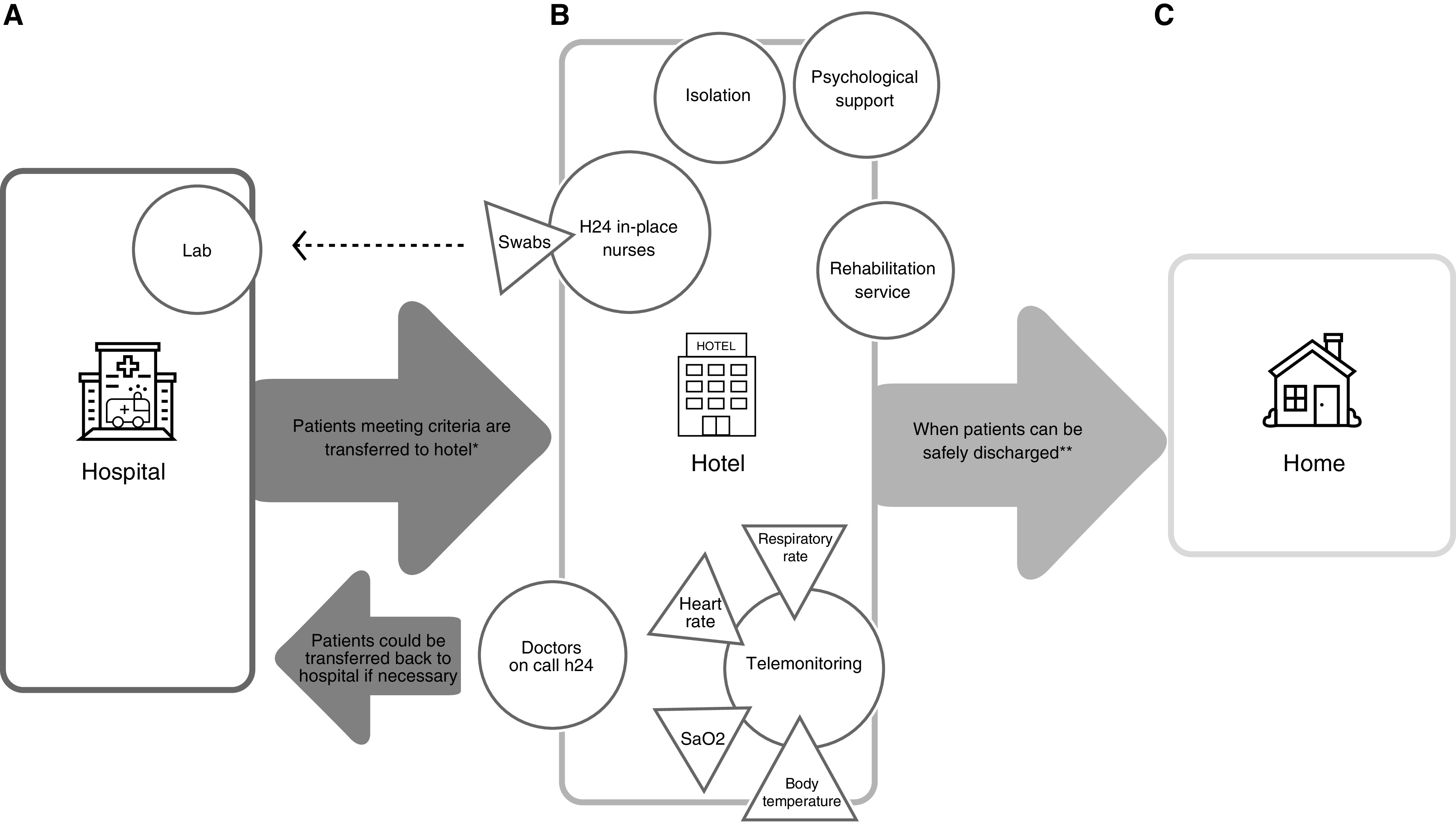
Patient flow between the various sites highlighting crucial checkpoints and potential challenges. (*A*) The hospital adopts a “reverse triage” method to free up beds, discharging patients in order of greater clinical stability. Patients who need to continue isolation (when not possible at home, or when they live in a care home) or medical monitoring, or both, are transferred to the hotel facility if medical stability is met. The proximity between the hotel and the hospital allows for rapid delivery of nasopharyngeal swabs to the laboratory and patients to the emergency room if necessary. (*B*) Key functions provided in the hotel: isolation through accommodation in single rooms, telemonitoring, nurses present 24 h/d, doctors on call, psychological assistance, and physiotherapy services. (*C*) Once two consecutive negative swabs are obtained, the patient can go home safely, without the risk of contagion to family members or care home residents or their caregivers. *Patients with a positive severe acute respiratory syndrome coronavirus 2 (SARS-CoV-2) swab or a clinical diagnosis of coronavirus disease (COVID-19), clinically stable, with no need for oxygen therapy nor intravenous therapy, able to use a smartphone, independent in daily routine activities, and unable to maintain effective home isolation can be admitted. **Patients undergo SARS-CoV-2 swabs in the hotel after 48–72 hours (if positive) or after 24 hours (if negative). After two consecutive negative swabs, the patient is safely discharged from the hotel. Figure icons were made by catkuro, iconixar, and Good Ware from www.flaticon.com and are used by permission. H24 = 24 h/d.

Countries with strict social distancing measures in place have closed hotels, leaving a large number of hotel rooms empty. Safely using this large unused capacity with telemedicine-enabled monitoring for the above categories of patients with COVID-19 could release much-needed hospital beds and avert the tipping point at which hospitals become overwhelmed. Telemonitoring, namely remote monitoring of patients by healthcare personnel who, through dedicated tools, are able to monitor the vital parameters and symptoms of the patients, has already been used for other respiratory diseases (3). In our model (Figure 1), Sa_O_2__, heart rate, body temperature, and respiratory rate are checked by respiratory physicians twice daily. Telephone help-desk support is available to patients, including direct contact with hotel nurses (or physicians, as appropriate) 24 hours a day. If deemed necessary, the physician visits the patient and, if needed, can alert the emergency room of the nearby hospital. Given the acute shortage of healthcare workers, our new model allows doctors to monitor many patients efficiently. The model also enables efficient use of nursing care, given the adjacency of rooms in corridors, as well as more efficient use of staff time and personal protective equipment, compared with the alternative of visiting geographically dispersed patients in their homes. Crucially, given the self-contained nature of hotel rooms with their *en suite* bathroom facilities, patients can be cared for and monitored in isolation with no risk of transmitting infection to vulnerable patients and healthcare workers (which would be the risk of staying in hospital) or to their cohabitants at home or in a residential care home (if discharged prematurely). Other on-site personnel are necessary: a pharmacist takes care of drugs and material supplies, the administrative staff receives requests for rooms from the hospital and manages the logistics, and a physiotherapist is available to let the hosts perform the necessary rehabilitation after a prolonged hospitalization. Room cleaning is performed by the hospital cleaning staff, who are already trained in sterilization of contaminated spaces.

The main reception area in the hotel lobby allows for surveillance to ensure orderly delivery of supplies and prevent access by visitors, and the existing hotel kitchens are used to prepare patients’ meals. Even physicians and nurses affected by COVID-19 and requiring isolation could take advantage of hotel accommodations to protect family members from infection. Either a data network or the Wi-Fi provided by the hotel can be used to send parameters to the telemonitoring platform at specified times and to easily connect to the physician, when necessary. Remote-monitoring service companies currently provide disposable kits to measure pulse oximetry and body temperature; in the future, national healthcare systems should consider investing in these devices to promote systematic telemonitoring programs (4, 5).

## Criteria for Admission

Clinically stable patients (in the absence of fever and with other parameters in order, without relevant symptoms) with resolving COVID-19 not requiring oxygen or intravenous therapies who are able to use a smartphone and who cannot maintain effective home isolation are eligible for continuing isolation in telemonitored, repurposed hotel rooms.

## Criteria for Medical Escalation or Discharge

Patients can be discharged from the hotel in one of two situations. A deterioration in clinical condition, detected via telemonitoring (e.g., increasing respiratory rate, declining Sa_O_2__) and confirmed by a visit to the patient by a nurse or doctor, mandates readmission to the hospital. Clinical recovery associated with two serial PCR-negative swab results enables discharge to the home.

## Limitations

The main potential limitations of hotel-based treatment of patients with COVID-19 are logistical. Dedicated access routes for patients and staff need to be delineated, ensuring the best possible infection prevention. A hotel may have furnishings, such as carpet, that make room cleaning less straightforward than cleaning hospital floors, and cleaning staff must therefore be properly trained to sanitize the rooms and dispose of telemonitoring kits appropriately (4). Hotel management needs to discuss and agree on the costs related to the management of patients with COVID-19 and the protection of the hotel staff. In our experience, the costs were kept particularly low by the combination of the complete absence of tourists and the willingness to contribute to the solution to the pandemic situation. In our particular case, charity support was also provided (*see* first-page footnote). A decontamination protocol at the end of the patient stay is expected. Furthermore, it is necessary to recognize the diagnostic and therapeutic requirements that patients just discharged from the hospital, still PCR-positive for severe acute respiratory syndrome coronavirus 2 (SARS-CoV-2), may have. Many diagnostic tests and therapies are not feasible in a hotel environment, but, in any case, hotels close to a hospital should be prioritized for this model. Apart from evaluation of parameters and emergency management, the liability for the patients remains the responsibility of the general practitioner, who then takes care of any drug prescriptions and the issue of certificates. Currently in our model, only telephone calls are available, but it could be useful to improve the service by using video calls, allowing a more accurate examination by the physicians and greater confidence for the patient. In this regard, the psychological well-being of patients must be considered; some patients may experience a sense of loneliness owing to the absence of family members (compared with when discharged straight to the home) or the less frequent encounters with nurses and doctors (compared with remaining in an isolation room in a hospital) (6, 7). In our model, a psychological support service has been set up for patients who needed it.

## Feasibility of the Model

Currently, this model has been launched in Rome, Italy, by Fondazione Policlinico Universitario A. Gemelli in collaboration with the Lazio Region in a hotel adjacent to the hospital. Italy, the first European country to suffer a high rate of infection and mortality from SARS-CoV-2, needed to identify a new model of healthcare and social reorganization (1, 8). Major challenges have included quickly informing citizens about implementing new anticontagion and self-isolation rules, as well as redistributing patients with COVID-19 to dedicated wards and building new ICUs.

In the 30-day period between April 1 and May 1, 239 patients have been hosted, of whom 126 have been discharged to the home without any complications or adverse events after a median duration of stay of 12 days (range, 3–23 d). None of the patients were hospitalized after discharge from the hotel. This model, moreover, provides a much-needed source of revenue for hotels at zero occupancy and has been welcomed by the hotel industry in Rome. Our experience to date indicates that the model is safe, is acceptable to patients, and addresses a large unmet need. Hospitals with nearby suitable hotels may therefore consider adopting this model to help them meet the surging demand during the current pandemic, during the possible COVID-19 “second wave,” or during future situations in which a large-scale request for beds could be necessary. Moreover, an increasingly concrete use of telemedicine coordinated by respiratory physicians could represent a key tool for the long-term care of patients with COVID-19 and for other applications beyond COVID-19 treatment.

## Supplementary Material

Supplements

Author disclosures
