# Supplementary material for: Telemedicine-enabled Accelerated Discharge of Patients Hospitalized with COVID-19 to Isolation in Repurposed Hotel Rooms
Source: Am J Respir Crit Care Med. 2020 Aug 15;202(4):508–10. doi: 10.1164/rccm.202004-1238OE (PMC7427391; doi:10.1164/rccm.202004-1238OE)
Supplement: Supplements [file rccm.202004-1238OE.html]

Telemedicine-enabled Accelerated Discharge of Patients Hospitalized with COVID-19 to Isolation in Repurposed Hotel Rooms | American Journal of Respiratory and Critical Care Medicine

- disclosures.pdf (164 KB)
